# An unusual case of Idelalisib‐related pneumonitis with severe respiratory failure

**DOI:** 10.1002/jha2.123

**Published:** 2020-10-26

**Authors:** Domingos Sousa, Maria Eduarda Couto, Alda Tavares, Teresa Ribeiro, Fabiana Santos Muñoz, Nelson Domingues, Isabel Oliveira, Mário Mariz

**Affiliations:** ^1^ Internal Medicine Department Centro Hospitalar e Universitário do Algarve, E.P.E. Portimão Portugal; ^2^ Onco‐hematology Department Instituto Português de Oncologia do Porto, y F.G., E.P.E. Porto Portugal; ^3^ Medical Oncology Department, Hospital Pedro Hispano Matosinhos Local Health Unit Matosinhos Portugal

Nonspecific idiopathic interstitial pneumonias (NSIP), commonly described as pneumonitis, have an extensive differential diagnosis. The introduction of new drugs to treat chronic lymphocytic leukemia (CLL) improved the therapeutic arsenal and prognosis. Idelalisib has been shown to be effective in controlling CLL. An acute onset of cough, dyspnoea, and fever are pneumonitis main manifestations, establishing a differential diagnosis with viral and bacterial respiratory infections [1, 2, 3].

A 67‐year‐old woman with a history of CLL since 2008 and multiple therapeutic lines was proposed for idelalisib, being admitted 12 months later for polyarthralgia, cough fever, and dyspnoea. On day 3 of admission, the patient develops severe respiratory failure (PaO_2_/FiO_2_ ratio = 150) and a markedly raised C‐reactive protein. The chest X‐ray showed bilateral interstitial infiltrates dispersed (Figure 1A) and the computed tomography revealed diffuse bilateral groundglass opacities (Figure 1B). Infectious causes have been excluded with multiple studies and bronchoalveolar lavage was negative for bacteria and fungus. Thus, idelalisib was stopped and methylprednisolone 500 mg for 3 days was started, tapered to 60 mg prednisolone with gradual weaning up during 6 weeks with complete recovery (Figure 2).

**FIGURE 1:**
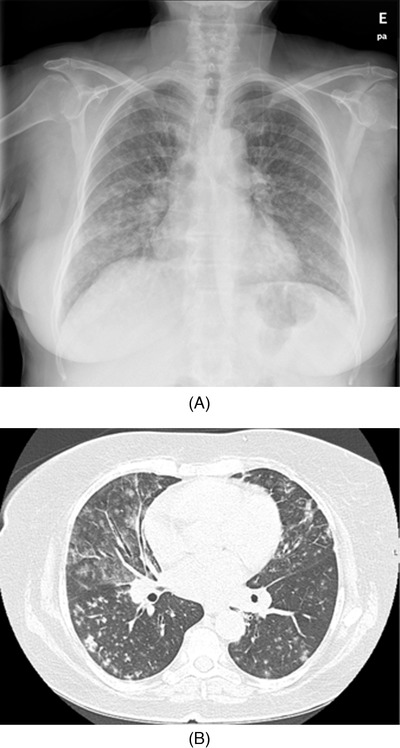
(A) Chest X‐ray shows bilateral interstitial infiltrates. (B) Thorax CT with diffuse bilateral ground glass opacities with micronodules

**FIGURE 2:**
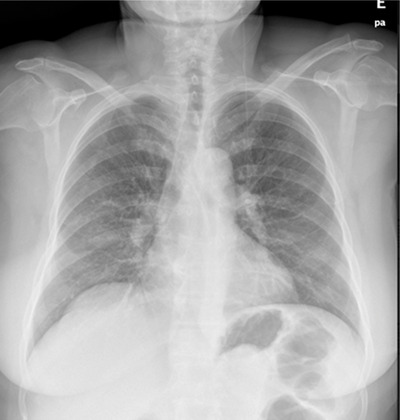
Chest X‐ray 1 week after treatment start shows improvement of the interstitial infiltrates

Idelalisib‐related pneumonitis may manifest late after treatment and lead to severe respiratory dysfunction; nonetheless a prompt diagnosis made in a timely manner can lead to a favorable outcome with complete recovery [1, 2, 3].

## CONFLICT OF INTEREST

The authors declare that there is no conflict of interest.

## HUMAN AND ANIMAL RIGHTS

This article does not contain any study with human and animals performed by any of the authors.

## INFORMED CONSENT

Informed consent was signed by the patient.

## AUTHOR CONTRIBUTIONS

Domingos Sousa: Acquisition of data, clinical and imaging data review, literature review, and final manuscript writing. Maria Eduarda Couto, Alda Tavares, Teresa Ribeiro, Fabiana Santos Muñoz, Nelson Domingues, Isabel Oliveira, and Mário Mariz: Important intellectual contribution and final manuscript writing.
